# 2,4-Bis(2-methoxy­phenyl)-3-aza­bicyclo­[3.3.1]nonan-9-one

**DOI:** 10.1107/S1600536809018686

**Published:** 2009-05-23

**Authors:** P. Parthiban, V. Ramkumar, Min Sung Kim, Se Mo Son, Yeon Tae Jeong

**Affiliations:** a Division of Image Science and Information Engineering, Pukyong National University, Busan 608 739, Republic of Korea; b Department of Chemistry, IIT Madras, Chennai 36, Tamil Nadu, India

## Abstract

In the title compound, C_22_H_25_NO_3_, the mol­ecule has a pseudo-mirror plane. The structure is a positional isomer of 2,4-bis(4-methoxy­phenyl)-3-aza­bicyclo­[3.3.1]nonan-9-one [Cox, McCabe, Milne & Sim (1985 ▶). *J. Chem. Soc. Chem. Commun.* pp. 626–628]. The 3-aza­bicyclo­[3.3.1]nonan-9-one moiety adopts a double chair conformation with equatorial orientations of both 2-methoxy­phenyl substituents on either side of the secondary amino group. The benzene rings are oriented at an angle of 33.86 (4)° with respect to each other and the meth­oxy groups point towards the carbonyl group. The crystal structure is stabilized by intermolecular N—H⋯π inter­actions.

## Related literature

For prevalence and biological activities of 3-aza­bicyclo­nonan-9-ones, see: Hardick *et al.* (1996 ▶); Jeyaraman & Avila (1981 ▶); Barker *et al.* (2005 ▶). For similiar structures, see: Parthiban *et al.* (2008*a* ▶,*b* ▶); Cox *et al.* (1985 ▶). For ring-puckering parameters, see: Cremer & Pople (1975 ▶).
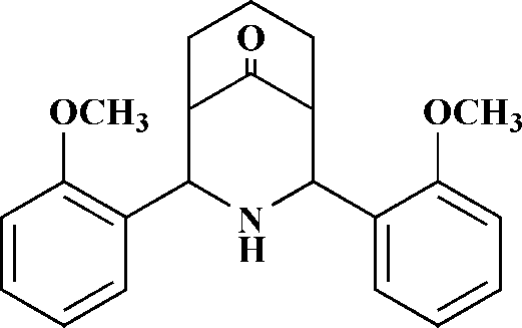


## Experimental

### 

#### Crystal data

C_22_H_25_NO_3_
*M* *_r_* = 351.43
Monoclinic, 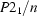

*a* = 7.8616 (2) Å
*b* = 20.8443 (6) Å
*c* = 11.4984 (3) Å
β = 95.670(10)°
*V* = 1874.37 (9) Å^3^
*Z* = 4
Mo *K*α radiation
μ = 0.08 mm^−1^
*T* = 298 K
0.58 × 0.42 × 0.35 mm

#### Data collection

Bruker APEXII CCD area-detector diffractometer
Absorption correction: multi-scan (*SADABS*; Bruker, 1999 ▶) *T*_min_ = 0.945, *T*_max_ = 0.972
14712 measured reflections
4527 independent reflections
3202 reflections with *I* > 2σ(*I*)
*R*_int_ = 0.023

#### Refinement

*R*[*F* ^2^ > 2σ(*F* ^2^)] = 0.048
*wR*(*F* ^2^) = 0.135
*S* = 1.03
4527 reflections
241 parameters
H atoms treated by a mixture of independent and constrained refinement
Δρ_max_ = 0.17 e Å^−3^
Δρ_min_ = −0.25 e Å^−3^

### 

Data collection: *APEX2* (Bruker, 2004 ▶); cell refinement: *APEX2*; data reduction: *SAINT-Plus* (Bruker, 2004 ▶); program(s) used to solve structure: *SHELXS97* (Sheldrick, 2008 ▶); program(s) used to refine structure: *SHELXL97* (Sheldrick, 2008 ▶); molecular graphics: *ORTEP-3* (Farrugia, 1997 ▶); software used to prepare material for publication: *SHELXL97*.

## Supplementary Material

Crystal structure: contains datablocks global, I. DOI: 10.1107/S1600536809018686/bx2207sup1.cif

Structure factors: contains datablocks I. DOI: 10.1107/S1600536809018686/bx2207Isup2.hkl

Additional supplementary materials:  crystallographic information; 3D view; checkCIF report

## Figures and Tables

**Table 1:** N—H⋯π geometry (Å, ° )

*D*—H⋯*A*	*D*—H	H⋯*A*	*D*⋯*A*	*D*—H⋯*A*
N1—H1*A*⋯*Cg*1^i^	0.90 (4)	2.75 (4)	3.58 (5)	152.87 (3)

Symmetry code: (i) 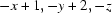. *Cg*1 is the centroid of the C9–C14 ring.

**Fig. 1.**
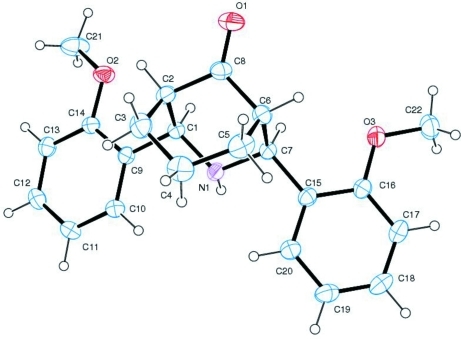
View of the title molecule with atoms represented as 30% probability ellipsoids.

**Fig. 2.**
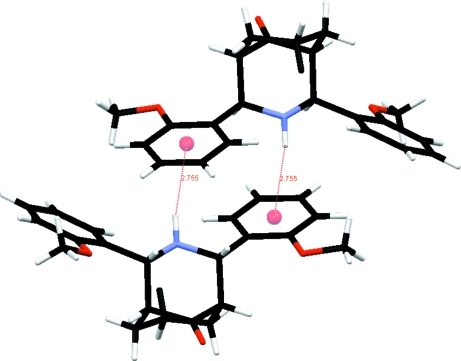
The N—H···π interactions.

## supplementary crystallographic information

### Comment

Widespread interest in the chemistry of 3-azabicyclononan-9-ones is due to their presence in numerous naturally occurring alkaloids and broad spectrum biological activities (Jeyaraman & Avila, 1981; Hardick *et al.*, 1996; Barker *et al.*, 2005). Since the stereochemistry plays a crucial role in eliciting the biological response, it is immense to establish the stereochemistry of the biologically important molecules. Even though similar compounds show double chair conformation (Parthiban *et al.*, 2008a,b), we have carried out the single-crystal XRD study for the title compound to know the impact of comparatively bulkier methoxy substituent on *ortho* position of the phenyl group, attached on either side of the secondary amino group.The molecule has a pseudo mirror plane.The structure is a positional isomer of 2,4-Bis(p-methoxyphenyl)-3-azabicyclo(3.3.1)nonan-9-one (Cox *et al.*, 1985). The title compound C_22_H_25_NO_3_, exists in double chair conformation with an equatorial orientation of the *ortho*-methoxyphenyl group on both sides of the secondary amino group with the torsion angle of C8—C2—C1—C9 and C8—C6—C7—C15 are -179.66 (3) and -179.76 (4)°, respectively. In both aryl groups, the methoxy substituent point towards the carbonyl group at an angle of 33.86 (4)° to each other. A study of torsion angles, asymmetry parameters and least-squares plane calculation shows that the piperidine ring adopts near ideal chair conformation with the deviation of ring atoms N1 and C8 from the C1/C2/C6/C7 plane by -0.641 (3) and 0.718 (3) Å, respectively; Q_T_ = 0.6101 (15) Å, q(2)= 0.0490 (15) Å, q(3)= -0.6081 (15) Å, θ = 175.41 (14)° (Cremer & Pople, 1975) whereas the cyclohexane ring atoms C4 and C8 deviate from the C2/C3/C5/C6 plane by -0.537 (4) and 0.710 (3) Å, respectively; Q_T_ = 0.5528 (17) Å, q(2)= 0.1286 (17) Å, q(3)= -0.5376 (17) Å, θ = 166.55 (18)°.

### Experimental

A mixture of cyclohexanone (4.90 g) and *ortho*-methoxybenzaldehyde (13.62 g) was added to a warm solution of ammonium acetate (5.78 g) in 50 ml of absolute ethanol. The mixture was gently warmed with stirring till the yellow color was formed during the mixing of the reactants and then allowed to stirring at room temperature up to the formation of product. At the end, the crude azabicyclic ketone was separated by filtration and washed with 1:5 ethanol–ether mixture till the solid became colorless. Recrystallization of the compound from ethanol gave X-ray diffraction quality crystals of 2,4-bis(2-methoxyphenyl)-3-azabicyclo[3.3.1]nonan-9-one.

### Refinement

Nitrogen H atoms were located in a difference Fourier map and refined isotropically. Other H atoms were fixed geometrically and allowed to ride on the parent C atoms,with aromatic C—H = 0.93 Å, aliphatic C—H = 0.98 Å and methylen C—H = 0.97 Å. The displacement parameters were set for phenyl, methylen and aliphatic H atoms at *U*_iso_(H) = 1.2*U*_eq_(C).

### Crystal data

**Table:**

C_22_H_25_NO_3_	*F*(000) = 752
*M**_r_* = 351.43	*D*_x_ = 1.245 Mg m^−^^3^
Monoclinic, *P*2_1_/*n*	Mo *K*α radiation, λ = 0.71073 Å
Hall symbol: -P 2yn	Cell parameters from 4251 reflections
*a* = 7.8616 (2) Å	θ = 2.6–27.0°
*b* = 20.8443 (6) Å	µ = 0.08 mm^−^^1^
*c* = 11.4984 (3) Å	*T* = 298 K
β = 95.867 (1)°	Block, colourless
*V* = 1874.37 (9) Å^3^	0.58 × 0.42 × 0.35 mm
*Z* = 4	

### Data collection

**Table:**

Bruker APEXII CCD area-detector diffractometer	4527 independent reflections
Radiation source: fine-focus sealed tube	3202 reflections with *I* > 2σ(*I*)
graphite	*R*_int_ = 0.023
φ and ω scans	θ_max_ = 28.3°, θ_min_ = 2.0°
Absorption correction: multi-scan (*SADABS*; Bruker, 1999)	*h* = −9→10
*T*_min_ = 0.945, *T*_max_ = 0.972	*k* = −27→26
14712 measured reflections	*l* = −15→13

### Refinement

**Table:**

Refinement on *F*^2^	Primary atom site location: structure-invariant direct methods
Least-squares matrix: full	Secondary atom site location: difference Fourier map
*R*[*F*^2^ > 2σ(*F*^2^)] = 0.048	Hydrogen site location: inferred from neighbouring sites
*wR*(*F*^2^) = 0.135	H atoms treated by a mixture of independent and constrained refinement
*S* = 1.03	*w* = 1/[σ^2^(*F*_o_^2^) + (0.065*P*)^2^ + 0.3026*P*] where *P* = (*F*_o_^2^ + 2*F*_c_^2^)/3
4527 reflections	(Δ/σ)_max_ = 0.001
241 parameters	Δρ_max_ = 0.17 e Å^−^^3^
0 restraints	Δρ_min_ = −0.25 e Å^−^^3^

### Special details

**Table:**

Geometry. All e.s.d.'s (except the e.s.d. in the dihedral angle between two l.s. planes) are estimated using the full covariance matrix. The cell e.s.d.'s are taken into account individually in the estimation of e.s.d.'s in distances, angles and torsion angles; correlations between e.s.d.'s in cell parameters are only used when they are defined by crystal symmetry. An approximate (isotropic) treatment of cell e.s.d.'s is used for estimating e.s.d.'s involving l.s. planes.
Refinement. Refinement of *F*^2^ against ALL reflections. The weighted *R*-factor *wR* and goodness of fit *S* are based on *F*^2^, conventional *R*-factors *R* are based on *F*, with *F* set to zero for negative *F*^2^. The threshold expression of *F*^2^ > σ(*F*^2^) is used only for calculating *R*-factors(gt) *etc*. and is not relevant to the choice of reflections for refinement. *R*-factors based on *F*^2^ are statistically about twice as large as those based on *F*, and *R*-factors based on ALL data will be even larger.

### Fractional atomic coordinates and isotropic or equivalent isotropic displacement parameters (Å^2^)

**Table:**

	*x*	*y*	*z*	*U*_iso_*/*U*_eq_	
C1	0.72892 (16)	0.98913 (6)	0.17176 (12)	0.0343 (3)	
H1	0.7974	0.9851	0.1055	0.041*	
C2	0.85201 (18)	1.00058 (7)	0.28460 (14)	0.0417 (3)	
H2	0.9208	1.0389	0.2735	0.050*	
C3	0.7636 (2)	1.00894 (8)	0.39684 (15)	0.0531 (4)	
H3A	0.8479	1.0232	0.4589	0.064*	
H3B	0.6781	1.0424	0.3840	0.064*	
C4	0.6779 (2)	0.94870 (9)	0.43724 (15)	0.0582 (4)	
H4A	0.5702	0.9424	0.3894	0.070*	
H4B	0.6529	0.9545	0.5175	0.070*	
C5	0.7881 (2)	0.88909 (8)	0.42979 (15)	0.0552 (4)	
H5A	0.7179	0.8515	0.4390	0.066*	
H5B	0.8775	0.8896	0.4945	0.066*	
C6	0.87146 (18)	0.88257 (7)	0.31544 (14)	0.0435 (4)	
H6	0.9521	0.8466	0.3229	0.052*	
C7	0.74651 (17)	0.87292 (6)	0.20328 (13)	0.0366 (3)	
H7	0.8145	0.8689	0.1368	0.044*	
C8	0.96882 (18)	0.94334 (7)	0.29817 (14)	0.0431 (4)	
C9	0.60420 (16)	1.04380 (6)	0.14722 (12)	0.0336 (3)	
C10	0.43367 (18)	1.03906 (7)	0.16792 (13)	0.0415 (3)	
H10	0.3947	1.0014	0.1994	0.050*	
C11	0.32021 (19)	1.08937 (8)	0.14269 (15)	0.0478 (4)	
H11	0.2061	1.0851	0.1560	0.057*	
C12	0.37750 (19)	1.14550 (7)	0.09793 (14)	0.0467 (4)	
H12	0.3019	1.1794	0.0818	0.056*	
C13	0.54701 (18)	1.15209 (7)	0.07648 (13)	0.0422 (3)	
H13	0.5850	1.1903	0.0462	0.051*	
C14	0.65935 (17)	1.10152 (6)	0.10043 (12)	0.0352 (3)	
C15	0.63790 (17)	0.81319 (6)	0.20774 (13)	0.0372 (3)	
C16	0.69939 (18)	0.75399 (6)	0.17200 (13)	0.0402 (3)	
C17	0.5962 (2)	0.69992 (7)	0.16825 (15)	0.0500 (4)	
H17	0.6372	0.6609	0.1436	0.060*	
C18	0.4323 (2)	0.70404 (8)	0.20125 (16)	0.0547 (4)	
H18	0.3631	0.6677	0.1982	0.066*	
C19	0.3707 (2)	0.76145 (8)	0.23863 (15)	0.0515 (4)	
H19	0.2610	0.7639	0.2618	0.062*	
C20	0.47349 (18)	0.81559 (7)	0.24150 (14)	0.0440 (4)	
H20	0.4314	0.8543	0.2666	0.053*	
C21	0.8836 (2)	1.15648 (10)	0.01693 (18)	0.0661 (5)	
H21A	0.8196	1.1579	−0.0587	0.099*	
H21B	1.0030	1.1519	0.0079	0.099*	
H21C	0.8656	1.1955	0.0583	0.099*	
C22	0.9277 (2)	0.69656 (8)	0.09613 (18)	0.0615 (5)	
H22A	0.9330	0.6641	0.1556	0.092*	
H22B	1.0402	0.7041	0.0736	0.092*	
H22C	0.8538	0.6825	0.0293	0.092*	
N1	0.63559 (14)	0.92907 (5)	0.18328 (11)	0.0358 (3)	
O1	1.12212 (14)	0.94572 (6)	0.29471 (13)	0.0676 (4)	
O2	0.82804 (12)	1.10343 (5)	0.08112 (10)	0.0470 (3)	
O3	0.86248 (14)	0.75428 (5)	0.14027 (11)	0.0532 (3)	
H1A	0.567 (2)	0.9222 (7)	0.1162 (15)	0.046 (4)*	

### Atomic displacement parameters (Å^2^)

**Table:**

	*U*^11^	*U*^22^	*U*^33^	*U*^12^	*U*^13^	*U*^23^
C1	0.0332 (6)	0.0317 (6)	0.0388 (8)	−0.0012 (5)	0.0068 (6)	−0.0005 (5)
C2	0.0361 (7)	0.0370 (7)	0.0508 (9)	−0.0062 (6)	−0.0013 (6)	−0.0005 (6)
C3	0.0589 (10)	0.0535 (9)	0.0449 (10)	0.0039 (8)	−0.0036 (7)	−0.0115 (7)
C4	0.0631 (11)	0.0733 (12)	0.0390 (9)	−0.0011 (9)	0.0097 (8)	−0.0040 (8)
C5	0.0598 (10)	0.0600 (10)	0.0444 (10)	−0.0077 (8)	−0.0023 (8)	0.0127 (8)
C6	0.0362 (7)	0.0377 (7)	0.0556 (10)	0.0035 (6)	−0.0008 (7)	0.0060 (7)
C7	0.0354 (7)	0.0309 (6)	0.0446 (8)	−0.0010 (5)	0.0093 (6)	0.0003 (6)
C8	0.0344 (7)	0.0488 (8)	0.0448 (9)	−0.0028 (6)	−0.0019 (6)	0.0000 (7)
C9	0.0345 (7)	0.0327 (6)	0.0336 (8)	−0.0008 (5)	0.0029 (5)	−0.0025 (5)
C10	0.0378 (7)	0.0399 (7)	0.0477 (9)	−0.0026 (6)	0.0091 (6)	0.0008 (6)
C11	0.0347 (7)	0.0532 (9)	0.0567 (10)	0.0050 (7)	0.0110 (7)	−0.0022 (8)
C12	0.0453 (8)	0.0438 (8)	0.0507 (10)	0.0126 (7)	0.0038 (7)	−0.0008 (7)
C13	0.0473 (8)	0.0356 (7)	0.0433 (9)	0.0015 (6)	0.0033 (7)	0.0029 (6)
C14	0.0339 (7)	0.0374 (7)	0.0346 (7)	−0.0021 (6)	0.0041 (5)	−0.0008 (6)
C15	0.0397 (7)	0.0348 (7)	0.0371 (8)	−0.0029 (6)	0.0031 (6)	0.0040 (6)
C16	0.0436 (8)	0.0344 (7)	0.0431 (9)	−0.0013 (6)	0.0060 (6)	0.0058 (6)
C17	0.0620 (10)	0.0343 (7)	0.0540 (10)	−0.0063 (7)	0.0073 (8)	0.0020 (7)
C18	0.0591 (10)	0.0470 (9)	0.0577 (11)	−0.0202 (8)	0.0045 (8)	0.0071 (8)
C19	0.0427 (8)	0.0565 (10)	0.0561 (10)	−0.0111 (7)	0.0089 (7)	0.0090 (8)
C20	0.0431 (8)	0.0427 (8)	0.0469 (9)	−0.0021 (6)	0.0086 (7)	0.0025 (7)
C21	0.0463 (9)	0.0827 (13)	0.0698 (13)	−0.0097 (9)	0.0081 (8)	0.0362 (10)
C22	0.0672 (11)	0.0458 (9)	0.0744 (13)	0.0096 (8)	0.0209 (9)	−0.0044 (8)
N1	0.0344 (6)	0.0299 (6)	0.0419 (7)	−0.0020 (5)	−0.0015 (5)	−0.0003 (5)
O1	0.0330 (6)	0.0683 (8)	0.1010 (11)	−0.0040 (5)	0.0034 (6)	0.0059 (7)
O2	0.0359 (5)	0.0468 (6)	0.0593 (7)	−0.0022 (4)	0.0104 (5)	0.0138 (5)
O3	0.0511 (6)	0.0345 (5)	0.0773 (8)	0.0022 (5)	0.0220 (6)	−0.0021 (5)

### Geometric parameters (Å, °)

**Table:**

C1—N1	1.4640 (16)	C11—C12	1.373 (2)
C1—C9	1.5110 (17)	C11—H11	0.9300
C1—C2	1.556 (2)	C12—C13	1.387 (2)
C1—H1	0.9800	C12—H12	0.9300
C2—C8	1.504 (2)	C13—C14	1.3846 (19)
C2—C3	1.537 (2)	C13—H13	0.9300
C2—H2	0.9800	C14—O2	1.3674 (16)
C3—C4	1.520 (2)	C15—C20	1.3879 (19)
C3—H3A	0.9700	C15—C16	1.4021 (19)
C3—H3B	0.9700	C16—O3	1.3684 (17)
C4—C5	1.522 (2)	C16—C17	1.3868 (19)
C4—H4A	0.9700	C17—C18	1.383 (2)
C4—H4B	0.9700	C17—H17	0.9300
C5—C6	1.534 (2)	C18—C19	1.376 (2)
C5—H5A	0.9700	C18—H18	0.9300
C5—H5B	0.9700	C19—C20	1.386 (2)
C6—C8	1.504 (2)	C19—H19	0.9300
C6—C7	1.552 (2)	C20—H20	0.9300
C6—H6	0.9800	C21—O2	1.4227 (18)
C7—N1	1.4636 (16)	C21—H21A	0.9600
C7—C15	1.5136 (17)	C21—H21B	0.9600
C7—H7	0.9800	C21—H21C	0.9600
C8—O1	1.2109 (17)	C22—O3	1.4216 (18)
C9—C10	1.3889 (18)	C22—H22A	0.9600
C9—C14	1.4044 (18)	C22—H22B	0.9600
C10—C11	1.388 (2)	C22—H22C	0.9600
C10—H10	0.9300	N1—H1A	0.907 (17)
			
N1—C1—C9	109.89 (10)	C11—C10—H10	119.3
N1—C1—C2	109.30 (11)	C9—C10—H10	119.3
C9—C1—C2	112.12 (11)	C12—C11—C10	119.60 (13)
N1—C1—H1	108.5	C12—C11—H11	120.2
C9—C1—H1	108.5	C10—C11—H11	120.2
C2—C1—H1	108.5	C11—C12—C13	120.61 (13)
C8—C2—C3	109.05 (13)	C11—C12—H12	119.7
C8—C2—C1	106.62 (11)	C13—C12—H12	119.7
C3—C2—C1	114.94 (12)	C14—C13—C12	119.63 (13)
C8—C2—H2	108.7	C14—C13—H13	120.2
C3—C2—H2	108.7	C12—C13—H13	120.2
C1—C2—H2	108.7	O2—C14—C13	123.71 (12)
C4—C3—C2	114.56 (13)	O2—C14—C9	115.45 (12)
C4—C3—H3A	108.6	C13—C14—C9	120.84 (12)
C2—C3—H3A	108.6	C20—C15—C16	118.20 (13)
C4—C3—H3B	108.6	C20—C15—C7	121.64 (12)
C2—C3—H3B	108.6	C16—C15—C7	120.09 (12)
H3A—C3—H3B	107.6	O3—C16—C17	123.81 (13)
C3—C4—C5	112.60 (14)	O3—C16—C15	115.77 (12)
C3—C4—H4A	109.1	C17—C16—C15	120.41 (13)
C5—C4—H4A	109.1	C18—C17—C16	119.98 (15)
C3—C4—H4B	109.1	C18—C17—H17	120.0
C5—C4—H4B	109.1	C16—C17—H17	120.0
H4A—C4—H4B	107.8	C19—C18—C17	120.47 (14)
C4—C5—C6	114.58 (13)	C19—C18—H18	119.8
C4—C5—H5A	108.6	C17—C18—H18	119.8
C6—C5—H5A	108.6	C18—C19—C20	119.51 (14)
C4—C5—H5B	108.6	C18—C19—H19	120.2
C6—C5—H5B	108.6	C20—C19—H19	120.2
H5A—C5—H5B	107.6	C19—C20—C15	121.41 (14)
C8—C6—C5	107.83 (13)	C19—C20—H20	119.3
C8—C6—C7	106.67 (12)	C15—C20—H20	119.3
C5—C6—C7	115.74 (12)	O2—C21—H21A	109.5
C8—C6—H6	108.8	O2—C21—H21B	109.5
C5—C6—H6	108.8	H21A—C21—H21B	109.5
C7—C6—H6	108.8	O2—C21—H21C	109.5
N1—C7—C15	109.52 (11)	H21A—C21—H21C	109.5
N1—C7—C6	110.07 (11)	H21B—C21—H21C	109.5
C15—C7—C6	112.93 (11)	O3—C22—H22A	109.5
N1—C7—H7	108.1	O3—C22—H22B	109.5
C15—C7—H7	108.1	H22A—C22—H22B	109.5
C6—C7—H7	108.1	O3—C22—H22C	109.5
O1—C8—C6	124.11 (14)	H22A—C22—H22C	109.5
O1—C8—C2	124.27 (14)	H22B—C22—H22C	109.5
C6—C8—C2	111.62 (12)	C7—N1—C1	113.70 (10)
C10—C9—C14	117.90 (12)	C7—N1—H1A	107.5 (10)
C10—C9—C1	122.25 (12)	C1—N1—H1A	108.7 (10)
C14—C9—C1	119.85 (11)	C14—O2—C21	117.45 (12)
C11—C10—C9	121.42 (13)	C16—O3—C22	118.24 (12)
			
N1—C1—C2—C8	58.24 (14)	C11—C12—C13—C14	0.0 (2)
C9—C1—C2—C8	−179.66 (11)	C12—C13—C14—O2	179.37 (14)
N1—C1—C2—C3	−62.72 (15)	C12—C13—C14—C9	−0.6 (2)
C9—C1—C2—C3	59.38 (15)	C10—C9—C14—O2	−179.60 (12)
C8—C2—C3—C4	−51.29 (18)	C1—C9—C14—O2	−0.42 (19)
C1—C2—C3—C4	68.33 (18)	C10—C9—C14—C13	0.4 (2)
C2—C3—C4—C5	43.6 (2)	C1—C9—C14—C13	179.54 (13)
C3—C4—C5—C6	−45.3 (2)	N1—C7—C15—C20	−26.68 (19)
C4—C5—C6—C8	54.13 (17)	C6—C7—C15—C20	96.37 (16)
C4—C5—C6—C7	−65.18 (18)	N1—C7—C15—C16	150.24 (13)
C8—C6—C7—N1	−57.03 (14)	C6—C7—C15—C16	−86.71 (16)
C5—C6—C7—N1	62.93 (15)	C20—C15—C16—O3	−179.66 (13)
C8—C6—C7—C15	−179.76 (11)	C7—C15—C16—O3	3.3 (2)
C5—C6—C7—C15	−59.81 (16)	C20—C15—C16—C17	1.4 (2)
C5—C6—C8—O1	117.71 (18)	C7—C15—C16—C17	−175.58 (13)
C7—C6—C8—O1	−117.36 (17)	O3—C16—C17—C18	−179.58 (15)
C5—C6—C8—C2	−62.84 (16)	C15—C16—C17—C18	−0.8 (2)
C7—C6—C8—C2	62.09 (16)	C16—C17—C18—C19	−0.4 (3)
C3—C2—C8—O1	−118.78 (18)	C17—C18—C19—C20	0.9 (3)
C1—C2—C8—O1	116.57 (17)	C18—C19—C20—C15	−0.2 (2)
C3—C2—C8—C6	61.77 (16)	C16—C15—C20—C19	−1.0 (2)
C1—C2—C8—C6	−62.88 (16)	C7—C15—C20—C19	176.01 (14)
N1—C1—C9—C10	17.27 (18)	C15—C7—N1—C1	−177.14 (11)
C2—C1—C9—C10	−104.49 (15)	C6—C7—N1—C1	58.14 (15)
N1—C1—C9—C14	−161.87 (12)	C9—C1—N1—C7	178.03 (11)
C2—C1—C9—C14	76.37 (16)	C2—C1—N1—C7	−58.54 (15)
C14—C9—C10—C11	0.4 (2)	C13—C14—O2—C21	−9.8 (2)
C1—C9—C10—C11	−178.73 (14)	C9—C14—O2—C21	170.19 (14)
C9—C10—C11—C12	−1.0 (2)	C17—C16—O3—C22	2.8 (2)
C10—C11—C12—C13	0.7 (2)	C15—C16—O3—C22	−176.03 (14)

### Hydrogen-bond geometry (Å, °)

**Table:**

*D*—H···*A*	*D*—H	H···*A*	*D*···*A*	*D*—H···*A*
N1—H1A···Cg1^i^	0.90 (4)	2.75 (4)	3.58 (5)	152.87 (3)

Symmetry codes: (i) −*x*+1, −*y*+2, −*z*.

## References

[bb1] Barker, D., Lin, D. H.-S., Carland, J. E., Chu, C. P.-Y., Chebib, M., Brimble, M. A., Savage, G. P. & McLeod, M. D. (2005). *Bioorg. Med. Chem.* **13**, 4565–4575. 10.1016/j.bmc.2005.04.054 15922601

[bb2] Bruker (1999). *SADABS* Bruker AXS Inc., Madison, Wisconsin, USA.

[bb3] Bruker (2004). *APEX2* and *SAINT-Plus* Bruker AXS Inc., Madison, Wisconsin, USA.

[bb4] Cox, P. J., McCabe, P. H., Milne, N. J. & Sim, G. A. (1985). *J. Chem. Soc. Chem. Commun.* pp. 626–628.

[bb5] Cremer, D. & Pople, J. A. (1975). *J. Am. Chem. Soc.* **97**, 1354–1358.

[bb6] Farrugia, L. J. (1997). *J. Appl. Cryst.* **30**, 565.

[bb7] Hardick, D. J., Blagbrough, I. S., Cooper, G., Potter, B. V. L., Critchley, T. & Wonnacott, S. (1996). *J. Med. Chem.* **39**, 4860–4866. 10.1021/jm9604991 8941400

[bb8] Jeyaraman, R. & Avila, S. (1981). *Chem. Rev.* **81**, 149–174.

[bb9] Parthiban, P., Ramkumar, V., Kim, M. S., Lim, K. T. & Jeong, Y. T. (2008*a*). *Acta Cryst.* E**64**, o1586. 10.1107/S160053680802268X PMC2962203 21203284

[bb10] Parthiban, P., Ramkumar, V., Kim, M. S., Son, S. M. & Jeong, Y. T. (2008*b*). *Acta Cryst.* E**64**, o2385. 10.1107/S1600536808037501 PMC2959882 21581356

[bb11] Sheldrick, G. M. (2008). *Acta Cryst.* A**64**, 112–122. 10.1107/S0108767307043930 18156677

